# Effects of creatine supplementation on lean mass, muscle size and muscle performance during immobilisation and rehabilitation: A systematic review

**DOI:** 10.1113/EP093965

**Published:** 2026-10-29

**Authors:** Jedd Pratt, Darren G. Candow, Eric S. Rawson, Scott C. Forbes, Konstantinos Prokopidis

**Affiliations:** ^1^ School of Sport and Exercise Sciences Manchester Metropolitan University Institute of Sport Manchester UK; ^2^ Faculty of Kinesiology and Health Studies University of Regina Regina Saskatchewan Canada; ^3^ Department of Health, Nutrition and Exercise Science Messiah University Mechanicsburg Pennsylvania USA; ^4^ Department of Physical Education Studies Brandon University Brandon Manitoba Canada; ^5^ Department of Musculoskeletal and Ageing Science, Institute of Life Course and Medical Sciences University of Liverpool Liverpool UK

**Keywords:** creatine, immobilisation, muscle atrophy, muscle disuse, rehabilitation

## Abstract

Creatine monohydrate (CrM) supplementation has established benefits in exercising populations; however, its efficacy during disuse and subsequent rehabilitation remains unclear. This systematic review assessed the effects of CrM supplementation on muscle size, lean mass and muscle performance (strength, power, endurance) during periods of disuse and rehabilitation. PubMed, Scopus, Web of Science and Cochrane Library were searched from inception to March 2026 following PRISMA guidelines. Six randomised placebo‐controlled trials were included (*n* = 118, mean ages: 15–24 years, disuse model: 1–2 weeks limb‐immobilisation). Two studies reported comparable reductions in quadriceps cross‐sectional area (CSA; assessed via CT or NMR imaging) between CrM and placebo during disuse (−5.6% vs. −5.5%; −11.0% vs. −9.4%), whereas two reported smaller reductions from CrM supplementation in lower‐limb lean mass (−5.6% vs. −8.9%) or preserved upper‐limb lean mass (0.9% vs. −3.7%) (assessed via bioelectrical impedance analysis (BIA) or dual energy X‐ray absorptiometry (DXA)). These contrasting findings may reflect limitations of BIA/DXA, including hydration effects, rather than preservation of muscle tissue. Effects on muscle performance were inconsistent, with favourable findings from CrM supplementation in upper‐ but not lower‐limb models. Four studies included recovery, three of which used resistance‐based rehabilitation. Two reported greater increases with CrM supplementation across quadriceps CSA (21.3% vs. 14.3%), lower‐limb lean mass (5.6% vs. 3.8%), power (52.2% vs. 35.2%) and strength (28.7% vs. 22.8%). Current evidence does not unanimously support CrM supplementation as an effective countermeasure to mitigate disuse‐induced muscle atrophy, lean mass loss or declines in muscle performance. Whether CrM supplementation meaningfully influences rehabilitation following disuse, particularly in older adults and clinical populations at greater risk of disuse atrophy and incomplete recovery, warrants further investigation.

## Abstract (highlights)

- **What is the topic of this review?**
  The effects of creatine monohydrate (CrM) supplementation on lean mass, muscle size and muscle performance (strength, power and endurance) during disuse and subsequent rehabilitation.
- **What advances does it highlight?**
  Current evidence does not support CrM supplementation as an effective countermeasure to mitigate disuse‐induced lean mass loss or muscle atrophy, and its effects on muscle performance during disuse are inconsistent. Limited evidence from resistance‐based disuse rehabilitation suggests that CrM supplementation may influence some measures of muscle size, lean mass and muscle performance during recovery, but the small and heterogeneous evidence base prevents firm conclusions regarding efficacy.

## 1 INTRODUCTION

Skeletal muscle is a central determinant of functional capacity, metabolic health and morbidity risk across the lifespan (Cheng et al., 2025; Kim & Kim, 2020; Wang et al., 2020). Preserving muscle mass and function is therefore critical for maintaining independence and reducing risk of chronic disease, particularly in ageing and clinical populations. Although skeletal muscle is highly plastic and capable of adapting positively and relatively rapidly to mechanical stimuli, this plasticity renders it particularly vulnerable to periods of disuse. Even short‐term unloading, such as that induced by bed rest, limb immobilisation or marked reductions in daily ambulation, leads to decreased muscle protein synthesis and accelerated muscle atrophy (Breen et al., 2013; Brook et al., 2022; Kortebein et al., 2007). These structural changes can be accompanied by impairments to neuromuscular function (Sarto et al., 2022), mitochondrial alterations (Noone et al., 2025) and insulin sensitivity (Mahmassani et al., 2019). Importantly, periods of disuse occur frequently in both clinical and free‐living contexts, as a consequence of hospitalisation, injury or illness. In older adults, repeated episodes of inactivity may worsen typical age‐related trajectories of muscle mass and strength and thereby increase the risk of sarcopenia and functional dependence (Pratt et al., 2025; Wall et al., 2013).

Given the prevalence and impact of disuse‐induced muscle atrophy, identifying strategies that can attenuate or prevent these losses is of considerable clinical and public health importance. While resistance exercise is the most potent stimulus for maintaining or restoring muscle mass (McKendry et al., 2024), it is often not feasible during periods of immobilisation or illness. Instead, nutritional strategies may represent a practical and scalable approach to support muscle health during disuse.

Creatine monohydrate (CrM) supplementation has been proposed as a potential nutritional strategy in this context due to its well‐established role in skeletal muscle energetics. The creatine (Cr)–phosphocreatine (PCr) system functions as a rapid buffer for ATP, facilitating energy provision during periods of increased metabolic demand (Wallimann et al., 1992). Beyond this primary role, CrM supplementation may also influence skeletal muscle through additional, although not yet fully understood, mechanisms. For example, CrM loading of skeletal muscle creates an osmotic gradient that leads to increased water retention and thus cellular swelling, which is believed to provide an anabolic stimulus for processes and pathways involved in muscle hypertrophy (Lang et al., 1998; Safdar et al., 2008). CrM supplementation may also affect anabolic growth and myogenic transcription factors (Chilibeck et al., 2017) and satellite cell activity, a key regulator of muscle regeneration and repair (Dangott et al., 2000; Olsen et al., 2006). In addition, CrM may influence aspects of neuromuscular function, with short‐term supplementation shown to improve force–velocity characteristics, muscle fibre conduction velocity and neuromuscular fatigue (Bazzucchi et al., 2009; Stout et al., 2007). However, whether these effects translate to the neuromuscular impairments observed during muscle disuse is unknown. There is also evidence that immobilisation‐induced disuse may reduce intramuscular PCr content (Fransen et al., 2015; Hespel et al., 2001; Luo et al., 2020; MacDougall et al., 1977) and lead to alterations in creatine transporter content (Luo et al., 2020). However, other studies have shown no significant change in PCr during disuse, but show alterations in phosphagen metabolism, including increased inorganic phosphate and impaired PCr recovery kinetics, alongside reductions in mitochondrial oxidative capacity (Pathare et al., 2008, 2005). It is therefore plausible that increasing intramuscular creatine availability through CrM supplementation may help preserve energy buffering capacity and, in turn, support skeletal muscle health during periods of disuse.

In exercising populations, CrM supplementation has been shown to augment improvements in lean mass (∼50% of lean mass is estimated to be skeletal muscle; Buckinx et al., 2018) and strength associated with resistance exercise (Wang et al., 2024). However, whether these effects translate to periods of disuse or rehabilitation following disuse remains unclear. This distinction is important as the efficacy of CrM supplementation may be contingent upon the presence of a mechanical stimulus (i.e., resistance exercise). Notably, the rehabilitation period following disuse represents a physiologically distinct condition from conventional resistance training, as skeletal muscle undergoing reloading has been exposed to unloading‐induced reductions in muscle mass, neuromuscular function and metabolic capacity. The reintroduction of mechanical loading and associated reactivation of anabolic signalling pathways may therefore provide a more favourable physiological environment for CrM supplementation to exert potential effects. Consequently, the extent of the effects of CrM supplementation during disuse rehabilitation may differ from those shown in healthy exercising populations without prior exposure to disuse.

To date, no systematic evidence synthesis has been completed in this area. Studies employing disuse models such as limb immobilisation have reported heterogeneous findings, with some showing attenuation of lean mass loss or muscle performance declines (Fransen et al., 2015; Johnston et al., 2009; Juhasz et al., 2018) and others showing minimal or no effect (Backx et al., 2017; Derave et al., 2003; Hespel et al., 2001). Such discordance may stem from differences in study design, including variation in the duration and severity of disuse, the timing and dosage of CrM supplementation, population characteristics and outcome measures assessed. A comprehensive appraisal is therefore required to characterise the existing evidence base, identify sources of heterogeneity and clarify whether CrM supplementation is an effective strategy for preserving skeletal muscle health during disuse and subsequent rehabilitation.

Accordingly, the aim of this study was to systematically evaluate the effects of CrM supplementation on muscle size, lean mass and muscle performance (i.e., strength, power, endurance) during periods of disuse and subsequent rehabilitation. Although disuse‐induced muscle atrophy is particularly relevant to clinical populations, studies involving participants with chronic medical conditions were excluded to reduce heterogeneity associated with disease‐specific metabolic and functional impairments and therefore isolate the effects of CrM supplementation.

## 2 METHODS

This systematic review was conducted in accordance with the Preferred Reporting Items for Systematic Reviews and Meta‐Analyses (PRISMA) guidelines (Page et al., 2021). The protocol was registered in the International Prospective Register of Systematic Reviews (PROSPERO) (CRD420261340025).

### 2.1 Search strategy

Two independent reviewers (J.P. and K.P.) searched PubMed, Scopus, Web of Science and Cochrane library from inception until March 2026. A comprehensive search strategy including the following terms: ‘creatine monohydrate AND (‘hypokinesia’ OR ‘hypoactivity’ OR ‘muscle disuse’ OR ‘disuse atrophy’ OR ‘immobiliz*’ OR ‘immobilis*’ OR ‘bed rest’ OR ‘bedridden’ OR ‘bedrest’ OR ‘head‐down tilt’) was used. A manual search of references cited in the selected articles and published reviews was also performed. Studies were included based on the following criteria: (1) must be a randomised controlled trial; (2) included CrM vs. placebo as intervention and comparator, respectively; (3) assessed muscle‐related outcomes in relation to muscle size, lean mass and performance; and (4) participants were healthy. Studies were excluded if they: (1) were non‐human trials; (2) included participants with chronic medical conditions; and (3) a full text was not available (i.e., conference abstract) and not in English.

### 2.2 Data extraction

Two authors (J.P. and K.P.) extracted data regarding the authors and date of publication, health status, study design, sample size, participant age and sex, treatment form, dose, and duration, muscle size, lean mass, muscle performance (i.e., strength, power, endurance), body composition assessment tool, and muscle performance assessment method.

### 2.3 Risk of bias assessment

Risk of bias in the included studies was assessed using the Cochrane Risk‐of‐bias 2 (RoB2) tool (Sterne et al., 2019) and evaluated by two independent reviewers (J.P. and K.P.). Appraisal of bias risk using the RoB2 tool included assessment of the domains of bias in randomized clinical trials: randomisation process; deviations from intended interventions; missing outcome data; measurement of the outcome; selection of the reported result. According to the RoB2 tool scoring system, overall risk‐of‐bias judgements were classified as low risk, some concerns, or high risk of bias.

### 2.4 Data synthesis

We employed a narrative synthesis due to the limited number of studies combined with heterogeneity across methodologies (e.g., disuse model, rehabilitation element, outcome measures, measurement technique).

## 3 RESULTS

The initial literature search yielded 100 publications. Following the exclusion of 66 duplicates, 34 unique publications were screened from which 24 were marked as ineligible after reading their titles and abstracts. In total, 10 full texts were assessed, but two were non‐human studies (Aoki et al., 2004; Crassous et al., 2009) and two had no outcomes of interest (Eijnde et al., 2005; Op ’t Eijnde et al., 2001). Overall, six studies were included in this systematic review (Backx et al., 2017; Derave et al., 2003; Fransen et al., 2015; Hespel et al., 2001; Johnston et al., 2009; Juhasz et al., 2018) (Figure 1).

**FIGURE 1:**
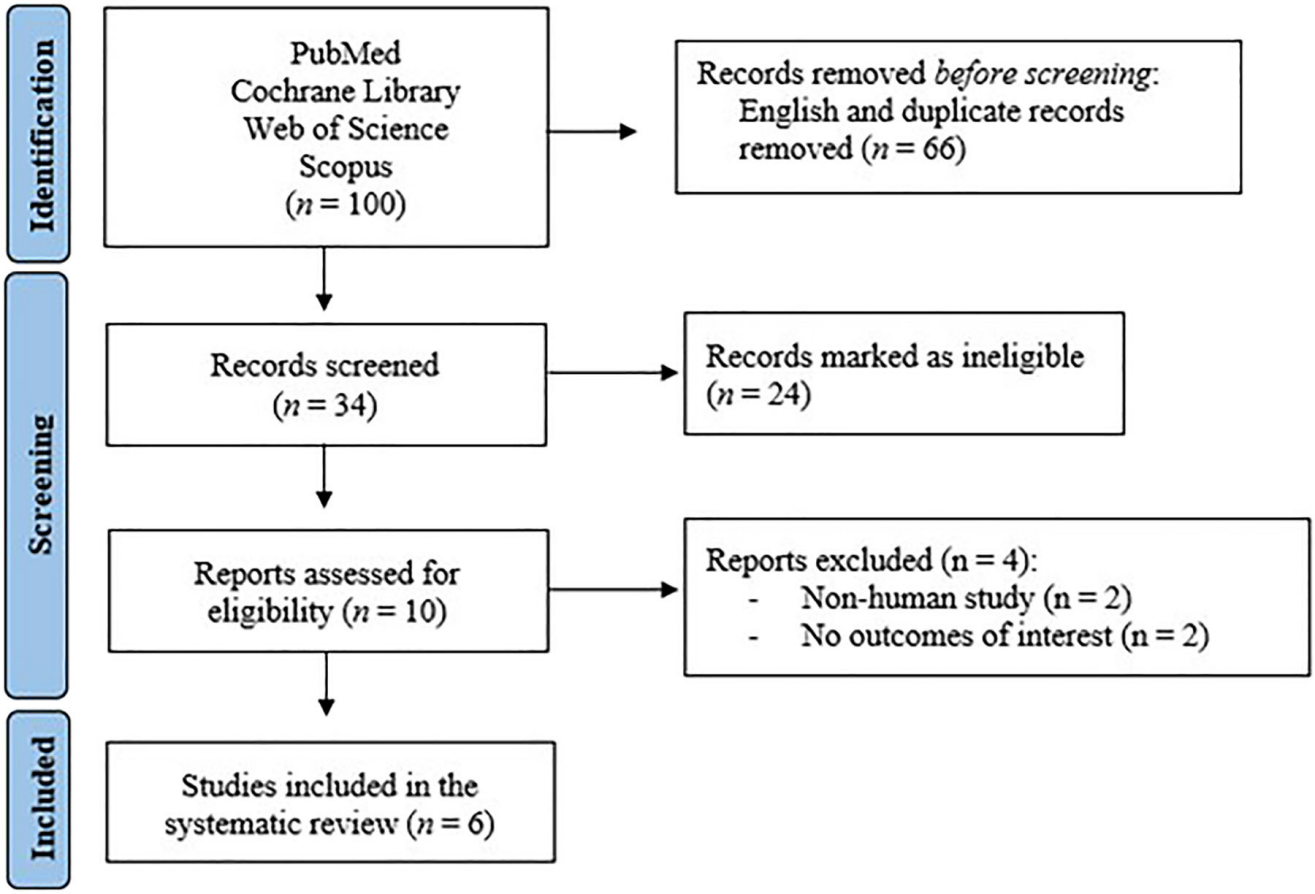
PRISMA flow chart of identification of studies.

Studies were published between 2001 and 2018 and were conducted in Europe and North America. Most studies employed a double‐blind, placebo‐controlled design (Backx et al., 2017; Derave et al., 2003; Fransen et al., 2015; Hespel et al., 2001; Juhasz et al., 2018), with one single‐blind crossover trial (Johnston et al., 2009). Sample sizes were relatively small, ranging from 7 to 27 participants, and predominantly young adults (ages between 15 and 24 years). All studies examined the effect of CrM supplementation during short‐term muscle disuse induced by immobilisation of the lower or upper limb (1–2 weeks). Three studies included a structured resistance training rehabilitation element ranging in length from 4 to 10 weeks (Derave et al., 2003; Hespel et al., 2001; Juhasz et al., 2018), and one study included a passive recovery phase for 1 week (Backx et al., 2017). CrM dosage typically consisted of 20 g/day for 5–7 days followed by a maintenance dose of between 3–5 g/day (in studies with a rehabilitation element), although protocols varied in duration and only one included a pre‐loading phase (Backx et al., 2017). Primary outcomes focused on muscle size, lean mass and muscle performance (i.e., strength, power, endurance). Muscle size was assessed through cross‐sectional area (CSA) obtained from computed tomography (CT) (Backx et al., 2017) or nuclear magnetic resonance (NMR) imaging (Hespel et al., 2001). Lean mass was assessed using dual energy X‐ray absorptiometry (DXA) (Johnston et al., 2009) or bioelectrical impedance analysis (BIA) (Juhasz et al., 2018). Muscle performance was assessed by one repetition maximum (1RM) testing (Backx et al., 2017; Johnston et al., 2009), dynamometry‐based strength, power, or endurance testing (Derave et al., 2003; Hespel et al., 2001; Johnston et al., 2009; Juhasz et al., 2018), or ergometry‐based endurance testing (Fransen et al., 2015). Characteristics of the included studies are presented in Table 1.

**TABLE 1:** Characteristics of included studies.

Study (author, year, country)	Design	Population (*n*, age, sex)	Disuse model	Intervention (dose, duration, pre‐loading [Y/N], rehabilitation [Y/N])	Control	Primary outcomes	Assessment methods
Backx et al. (2017) Netherlands	Double‐blind RCT	*n* = 27; 23 years; M only	Lower‐limb cast immobilisation (1 week)	CrM (5−20 g/day, 12 days, Y: 5 days, Y: 7 days of passive recovery)	Placebo	Muscle size, strength	CT, 1RM
Fransen et al. (2015) USA	Double‐blind RCT	*n* = 25; 24 years; M + F	Upper‐limb cast immobilisation (1 week)	CrM (20 g/day, 1 week, N, N)	Placebo	Endurance	Ergometry
Johnston et al. (2009) Canada	Single‐blind crossover RCT	*n* = 7; 22 years; M only	Upper‐limb cast immobilisation (2 × 1 week, 7 days apart)	CrM (20 g/day, 1 week, N, N)	Placebo	Lean mass, strength, endurance	DXA, 1RM, dynamometry
Hespel et al. (2001) Belgium	Double‐blind RCT	*n* = 19; 22 years; M + F	Lower‐limb cast immobilisation (2 weeks)	CrM (5−20 g/day, 2 weeks, N, Y: 10 weeks of progressive resistance training)	Placebo	Muscle size, strength, power	NMR imaging, dynamometry
Derave et al. (2003) Belgium	Double‐blind RCT	*n* = 22; 20 years; M + F	Lower‐limb cast immobilisation (2 weeks)	CrM (2.5−15 g/day, 2 weeks, N, Y: 6 weeks of progressive resistance training)	Placebo	Strength, endurance	Dynamometry
Juhasz et al. (2018) Hungary	Double‐blind RCT	*n* = 18; 15 years; M + F	Lower‐limb bandage immobilisation (2 weeks)	CrM (5−20 g/day, 2 weeks, N, Y: 4 weeks of progressive strength and balance training)	Placebo	Lean mass, strength	BIA, dynamometry

Abbreviations: CrM, creatine monohydrate; BIA, bioelectrical impedance analysis; CT, computed tomography; DXA, dual‐energy X‐ray absorptiometry; NMR, nuclear magnetic resonance; RCT, randomised controlled trial; RM, repetition maximum.

### 3.1 Effects of CrM supplementation during muscle disuse

#### 3.1.1 Muscle size and lean mass

Backx et al. (2017) and Hespel et al. (2001) reported comparable reductions in quadriceps CSA following 1 and 2 weeks of cast‐induced immobilisation with CrM supplementation compared with placebo (−5.6% vs. −5.5%, *P* = 0.76 and −11.0% vs. −9.4%, *P* = 0.30; respectively) (Table 2). In contrast, Johnston et al. (2009) demonstrated preservation of upper‐limb lean mass during 1 week of immobilisation with CrM supplementation (+0.9%) compared with a reduction of 3.7% in the placebo group (*P *< 0.05). Similarly, Juhasz et al. (2018) reported attenuated losses of lower‐limb lean mass during 2 weeks of immobilisation with CrM supplementation compared with placebo (−5.6% vs. −8.9%, *P *< 0.01).

**TABLE 2:** Effects of creatine monohydrate (CrM) supplementation on muscle outcomes during disuse and subsequent rehabilitation.

		Disuse phase			Rehabilitation phase		
Study	Muscle outcome	C(Δ% change)	Placebo (Δ% change)	Direction of effect	CrM(Δ% change)	Placebo (Δ% change)	Direction of effect
Backx et al. (2017)	Size (quadriceps CSA)	−5.6	−5.5	No effect	+1.1	+2.0	No effect
	Strength (knee extensor)	−9.2	−5.6	No effect	+3.8	0	No effect
Fransen et al. (2015)	Endurance (wrist flexor)	−7.5 to −11.6	−17.2 to −20	Favours CrM^*^	—	—	—
Johnston et al. (2009)	Mass (upper‐limb lean mass)	+0.9	−3.7	Favours CrM	—	—	—
	Strength (elbow flexor/extensor)	−4.1/−3.8	−21.5/−18.3	Favours CrM	—	—	—
	Endurance (elbow flexor/extensor)	−9.6/−6.5	−43.0/−35.0	Favours CrM	—	—	—
Hespel et al. (2001)	Size (quadriceps CSA)	−11.0	−9.4	No effect	+15.0 to +21.3	+9.2 to +14.3	Favours CrM
	Power (knee extensor)	−25.7	−23.8	No effect	+41.6 to +52.2	+27.8 to +35.2	Favours creatine
	Strength (knee extensor)	−20.6	−22.5	No effect	+37.5 to +50.0	+32.5 to +41.9	No effect
Derave et al. (2003)	Strength (knee extensor)	−28.9	−26.6	No effect	+45.8	+54.9	No effect
	Endurance (knee extensor)	−4.6	−5.1	No effect	+20.2	+7.8	Favours CrM
Juhasz et al. (2018)	Mass (leg lean mass)	−5.6	−8.9	Favours CrM	+1.9 to +5.6	+1.6 to +3.8	Favours CrM
	Strength (plantar flexors)	—	—	—	+10.4 to +28.7	+7.1 to +22.8	Favours CrM

Abbreviations: CSA, cross‐sectional area; —: not assessed at that time point.

* Trend *P* = 0.073.

#### 3.1.2 Muscle performance

Backx et al. (2017) showed CrM supplementation to have no effect on knee extensor 1RM (−9.2% CrM vs. −5.6% placebo, *P* = 0.20) (Table 2). Similar findings were also shown by Derave et al. (2003) and Hespel et al. (2001), who reported comparable declines in knee extensor strength, power and isometric torque between groups following 2 weeks of immobilisation (all *P *> 0.05). In contrast, Johnston et al. (2009) reported substantially smaller reductions in elbow flexor and extensor strength and endurance with CrM supplementation (−4.1% and −3.8%, −9.6% and −6.5%; *P *< 0.05) compared with placebo (−21.5% and −18.3%, −43.0% and −35.0%; *P *< 0.05). Fransen et al. (2015) showed that CrM supplementation led to lower, albeit not statistically significant (*P* = 0.073), reductions in wrist flexor endurance (−7.5 to −11.6%) compared with placebo (−17.2 to −20%).

### 3.2 Effects of CrM supplementation during disuse rehabilitation

#### 3.2.1 Muscle size and lean mass

Hespel et al. (2001) reported greater increases in quadriceps CSA during 3–10 weeks of rehabilitation in the CrM group (+15 to +21.3%) compared with placebo (+9.2 to +14.3%) (*P *< 0.05) (Table 2). Juhasz et al. (2018) also reported greater increases in leg lean mass during 2–4 weeks of rehabilitation with CrM supplementation (+1.9 to +5.6%) compared with placebo (+1.6 to +3.8%, *P *< 0.01). In contrast, Backx et al. (2017) observed no differences in quadriceps CSA gains between CrM and placebo (+1.1% vs. +2.0%, *P* = 0.52) during a short (7‐day), passive recovery period (i.e., no structured re‐training).

#### 3.2.2 Muscle performance

Hespel et al. (2001) demonstrated greater improvements in knee extensor power during 3–10 weeks rehabilitation in the CrM group (+41.6 to +52.2%) compared with placebo (+27.8 to +35.2%) (*P *< 0.05) (Table 2). Similarly, Derave et al. (2003) observed significant increases in knee extensor strength endurance with CrM supplementation (+20.2% vs. +7.8%, *P *< 0.05) during 6 weeks of rehabilitation, while Juhasz et al. (2018) reported greater improvements in plantar flexion torque during 2–4 weeks rehabilitation with CrM supplementation (+10.4 to +28.7% vs. +7.1 to +22.8%, *P *< 0.01). In contrast, Backx et al. (2017) showed no differences in knee extensor strength recovery between CrM and placebo (+3.8% vs. 0%, *P* = 0.32) following short‐term passive recovery.

### 3.3 Risk of bias

Four of the six included studies (Backx et al., 2017; Derave et al., 2003; Fransen et al., 2015; Hespel et al., 2001) were judged to have a low risk of bias across all five domains, resulting in an overall low risk of bias rating. In addition, two studies raised some concerns due to potential bias arising from deviations from the intended intervention and bias in the measurement of its outcomes (Johnston et al., 2009; Juhasz et al., 2018). Overall, the body of evidence was of low risk of bias, although caution is warranted when interpreting results from the two studies with some concerns (Figure 2).

**FIGURE 2:**
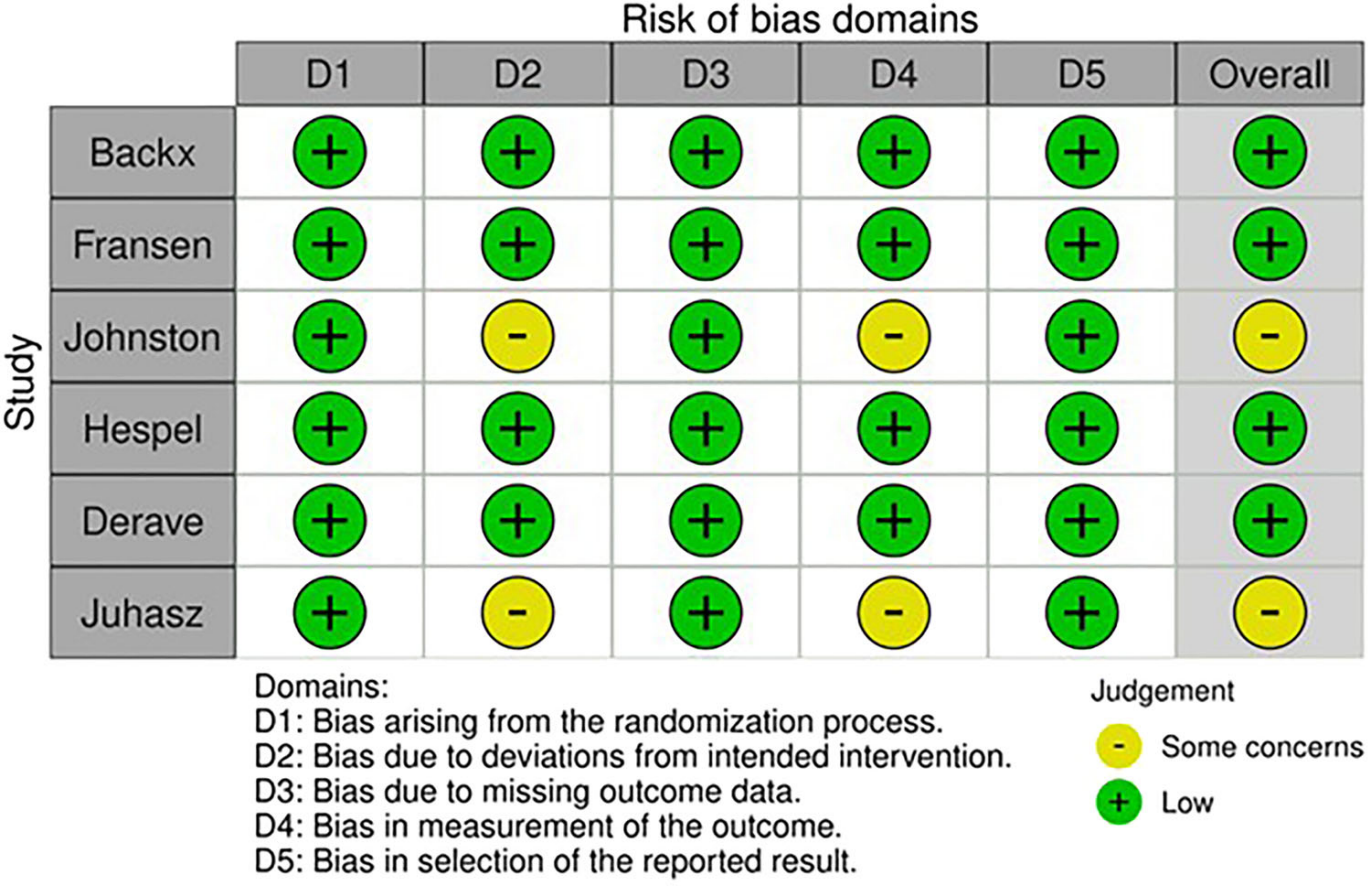
Risk of bias assessment of included studies.

## 4 DISCUSSION

This systematic review evaluated the effects of CrM supplementation on skeletal muscle size, lean mass and muscle performance (strength, power, endurance) during periods of disuse and subsequent rehabilitation. Overall, the available evidence does not support CrM supplementation as an effective countermeasure against muscle atrophy (CSA) during short‐term disuse, and findings for muscle performance are inconsistent. Evidence during rehabilitation following disuse is limited. Although some studies have reported greater recovery of muscle size, lean mass and selected measures of muscle performance with CrM supplementation when combined with resistance‐based rehabilitation, the small number of studies, modest sample sizes and methodological heterogeneity preclude firm conclusions regarding its efficacy in this setting.

### 4.1 Principal findings

#### 4.1.1 Disuse

The most consistent finding of this review was that CrM supplementation did not prevent the loss of muscle size (quadriceps CSA) during lower‐limb immobilisation. Both studies assessing muscle size reported similar reductions in quadriceps CSA between CrM and placebo groups (Backx et al., 2017; Hespel et al., 2001). These findings indicate that CrM supplementation does not prevent the loss of regional muscle tissue when mechanical loading is removed. In contrast, studies assessing regional lean mass reported smaller reductions during CrM supplementation compared with placebo (Johnston et al., 2009; Juhasz et al., 2018). However, these findings should not be interpreted as direct evidence of preserved skeletal muscle tissue. Studies reporting no benefit employed site‐specific measures of quadriceps CSA using NMR imaging or CT (Backx et al., 2017; Hespel et al., 2001), which provide high‐resolution quantification of localised morphological changes. In contrast, studies demonstrating an attenuated loss, or preservation of, limb lean mass relied upon regional DXA or BIA measures (Johnston et al., 2009; Juhasz et al., 2018). Although these methods are widely applied, estimates of lean mass can be influenced by fluctuations in fluid balance and cannot distinguish small changes in muscle mass from changes in other components of lean mass (e.g., water, organ weight) (Barreira & Tseh, 2020; Schierbauer et al., 2023). Accordingly, the favourable lean mass findings should be interpreted cautiously, particularly because they were not accompanied by comparable evidence from direct measures of muscle morphology.

Findings for muscle performance were similarly inconsistent. Studies involving lower‐limb immobilisation generally found no attenuation of reductions in strength, power or endurance with CrM supplementation (Backx et al., 2017; Derave et al., 2003; Hespel et al., 2001), whereas two studies employing upper‐limb disuse models reported smaller reductions in selected measures of muscle strength and endurance (Fransen et al., 2015; Johnston et al., 2009). Differences in the severity and duration of unloading, muscle groups studied, supplementation protocols and outcome measures may contribute to this heterogeneity. However, the limited number and small sample sizes of these studies prevent conclusions regarding whether the effects of CrM supplementation differ according to the disuse model or limb examined. Importantly, several studies reporting favourable findings were among the smallest included trials and were judged to have some concerns regarding risk of bias (Johnston et al., 2009; Juhasz et al., 2018), somewhat reducing confidence in these findings.

Overall, the available evidence does not show that CrM supplementation meaningfully attenuates muscle atrophy during short‐term disuse, while effects on muscle performance remain uncertain.

#### 4.1.2 Disuse rehabilitation

Of the four studies that assessed a subsequent recovery period (Backx et al., 2017; Derave et al., 2003; Hespel et al., 2001; Juhasz et al., 2018), only three incorporated structured resistance‐based rehabilitation (Derave et al., 2003; Hespel et al., 2001; Juhasz et al., 2018). Evidence regarding the effects of CrM supplementation during active recovery from disuse is therefore limited. Hespel et al. (2001) demonstrated greater increases in quadriceps CSA throughout 3–10 weeks of progressive resistance training with CrM supplementation compared with placebo, while Juhasz et al. (2018) reported greater recovery of leg lean mass during four weeks of reloading. These studies also reported greater improvements in selected measures of muscle strength, power or endurance with CrM supplementation. In contrast, Derave et al. (2003) reported similar recovery of maximal knee‐extensor torque between CrM and placebo following 6 weeks of resistance‐based rehabilitation, although a measure of fatigue recovery improved from baseline with CrM supplementation.

Collectively, these findings suggest that CrM supplementation may influence some aspects of muscle recovery during resistance‐based rehabilitation following disuse; however, the small number of studies and inconsistency between outcomes preclude firm conclusions. Moreover, the available studies include relatively small samples and differ in their rehabilitation protocols and methods for assessing muscle morphology and performance. Further studies are therefore required to clarify the efficacy and clinical relevance of CrM supplementation during active rehabilitation following disuse.

### 4.2 Mechanistic considerations

Several mechanisms have been proposed through which CrM supplementation could influence skeletal muscle during disuse or subsequent reloading. One plausible hypothesis is that CrM supplementation may influence metabolic responsiveness during periods of unloading. CrM supplementation has been reported to prevent reductions in muscle GLUT4 protein content during immobilisation and to augment the restoration of GLUT4 content and muscle glycogen repletion during disuse rehabilitation (Op ’t Eijnde et al., 2001). In a separate study conducted outside a disuse model, CrM supplementation reduced leucine oxidation by ∼20% and plasma leucine rate of appearance by ∼8% (indicator of muscle protein catabolism) compared to those on placebo (Parise et al., 2001). However, these findings have not been accompanied by consistent preservation of muscle size during disuse, suggesting that any metabolic effects of CrM supplementation are insufficient to prevent immobilisation‐induced muscle loss.

The effects of CrM supplementation may also differ once mechanical loading is reintroduced during rehabilitation. Importantly, these responses should not be viewed as directly equivalent to adaptations observed during conventional resistance training in non‐disused muscle, as prior unloading may alter the metabolic and anabolic recovery of skeletal muscle during reloading (Figueir et al., 2021; Franchi et al., 2025). Reloading following disuse reactivates key anabolic processes, including increases in myofibrillar protein synthesis, and restores neuromuscular function (Kilroe et al., 2025; Sarto et al., 2022). These processes increase metabolic demand within skeletal muscle, conditions under which the Cr–PCr system is functionally important. Moreover, CrM supplementation may augment anabolic signalling through cellular hydration mechanisms, given that increases in intracellular osmotic pressure have been suggested to support anabolism (Lang et al., 1998; Safdar et al., 2008). In addition, CrM supplementation may modulate insulin‐like growth factor I, myogenic transcription factors, satellite cell activity and markers related to the mechanistic target of rapamycin (mTOR) pathway (Chilibeck et al., 2017; Deldicque et al., 2005; Olsen et al., 2006), which may further support muscle hypertrophy and regeneration when combined with resistance exercise. However, these pathways have not been shown to mediate recovery following disuse, and CrM supplementation does not directly increase rates of myofibrillar protein synthesis (Antonio et al., 2025). The mechanisms underlying any potential effect of CrM supplementation during disuse rehabilitation therefore remain uncertain.

### 4.3 Limitations of the evidence and future research directions

Current knowledge is limited by the small number of studies and substantial methodological heterogeneity. Several trials included relatively small samples, and some of those reporting the largest apparent effects had concerns regarding risk of bias. Disuse models also varied in the limb studied, duration and severity of unloading, supplementation protocol, and rehabilitation strategy, limiting direct comparison between studies.

From a practical standpoint, current evidence does not support recommending CrM supplementation specifically to prevent lower‐body muscle atrophy or the reduction in lower‐body muscle performance during short‐term disuse. Evidence during rehabilitation is less certain. A small number of studies have reported greater improvements in muscle size, lean mass or selected performance outcomes when CrM supplementation was combined with resistance‐based rehabilitation, but these findings require replication in larger and methodologically robust trials before conclusions regarding efficacy or clinical relevance can be made. The current evidence base is also predominantly limited to healthy young adults, a population that typically demonstrates relatively rapid recovery following short‐term disuse. Therefore, the broader clinical relevance of CrM supplementation during disuse remains uncertain, particularly given the lack of studies in populations at greater risk of prolonged or incomplete recovery, such as older adults and clinical populations in whom muscle loss may be greater, recovery slower and the consequences of disuse more clinically important. Future trials should therefore include older adults and clinical populations, as well as other settings characterised by periods of unloading, such as spaceflight and elite sport.

Additionally, studies should incorporate mechanistic endpoints, such as muscle creatine content and biomarkers of anabolic signalling to better understand the underlying physiological pathways. There is also a need to further explore prehabilitation strategies, whereby CrM supplementation is initiated prior to planned immobilisation, to clarify whether CrM loading can attenuate the initial rapid phase of atrophy. However, Backx et al. (2017) was the only included study to initiate CrM loading before immobilisation (20 g/day for 5 days) and found no attenuation of quadriceps CSA or strength loss. This remained the case when analyses were restricted to participants whose muscle creatine content increased following supplementation (i.e., ‘responders’). Finally, broader application of high‐resolution imaging modalities and clinically relevant functional tests would improve comparability across studies and strengthen the overall evidence base. More consistent reporting of measurement error and minimal detectable changes within these tests would also aid interpretation of whether observed changes exceed measurement variability and reflect true physiological responses.

### 4.4 Conclusion

CrM supplementation does not appear to prevent or attenuate muscle atrophy during short‐term immobilisation‐induced disuse, and its effects on muscle performance are inconsistent. Limited evidence from resistance‐based disuse rehabilitation suggests that CrM supplementation may influence some measures of muscle size, lean mass and muscle performance during reloading, but the small and heterogeneous evidence base prevents firm conclusions regarding its efficacy. Further research is required to determine whether CrM supplementation meaningfully influences recovery following disuse and whether any effects extend to populations at greater risk of disuse‐related muscle decline.

## AUTHOR CONTRIBUTIONS

Jedd Pratt: data curation, formal analysis, methodology, writing—original draft. Darren G. Candow: conceptualisation, writing—review and editing. Eric S. Rawson: writing—review and editing. Scott C. Forbes: writing—review and editing. Konstantinos Prokopidis: conceptualisation, data curation, formal analysis, methodology, writing—original draft. All authors have read and approved the final version of this manuscript and agree to be accountable for all aspects of the work in ensuring that questions related to the accuracy or integrity of any part of the work are appropriately investigated and resolved. All persons designated as authors qualify for authorship, and all those who qualify for authorship are listed.

## CONFLICT OF INTEREST

D.G.C. has conducted industry‐sponsored research involving creatine supplementation and received creatine donations for scientific studies and travel support for presentations involving creatine supplementation at scientific conferences. In addition, D.G.C. serves as an Independent Scientific Advisory for Alzchem, Create, Infinis Nutrition, Iovate and Kiehl's (companies that manufacture creatine products) and as an expert witness/consultant in legal cases involving creatine supplementation. E.S.R. has conducted industry sponsored research involving creatine supplementation, has received creatine supplement donations for scientific studies and travel support for conference presentations involving creatine supplementation. In addition, E.S.R. serves on the Scientific Advisory Board for Alzchem (a company that manufactures creatine) and as an expert witness/consultant in legal cases involving creatine supplementation. S.C.F. has received creatine donations for scientific studies (Creapure), is a sports nutrition advisor for the International Society of Sports Nutrition (ISSN), and is a scientific advisor for Bear Balanced (a company which manufactures creatine products).

## FUNDING INFORMATION

None.

## GENERATIVE AI STATEMENT

No artificial intelligence tool was used for any element of this work.

## Data Availability

Data are available upon request to the corresponding author.
